# Longer Leukocyte Telomere Length Is Associated with Smaller Hippocampal Volume among Non-Demented *APOE* ε3/ε3 Subjects

**DOI:** 10.1371/journal.pone.0034292

**Published:** 2012-04-10

**Authors:** Mikael Wikgren, Thomas Karlsson, Johanna Lind, Therese Nilbrink, Johan Hultdin, Kristel Sleegers, Christine Van Broeckhoven, Göran Roos, Lars-Göran Nilsson, Lars Nyberg, Rolf Adolfsson, Karl-Fredrik Norrback

**Affiliations:** 1 Division of Psychiatry, Department of Clinical Sciences, Umeå University, Umeå, Sweden; 2 Department of Behavioral Sciences and Learning, Linköping University, Linköping, Sweden; 3 Department of Psychology, University of Oslo, Oslo, Norway; 4 Division of Clinical Chemistry, Department of Medical Biosciences, Umeå University, Umeå, Sweden; 5 Neurodegenerative Brain Diseases Group, Department of Molecular Genetics, VIB, Antwerp, Belgium; 6 Laboratory of Neurogenetics, Institute Born-Bunge, University of Antwerp, Antwerp, Belgium; 7 Division of Pathology, Department of Medical Biosciences, Umeå University, Umeå, Sweden; 8 Department of Psychology, Stockholm University, Stockholm, Sweden; 9 Stockholm Brain Institute, Stockholm, Sweden; 10 Department of Radiation Sciences (Radiology), Umeå University, Umeå, Sweden; 11 Department of Integrative Medical Biology (Physiology), Umeå University, Umeå, Sweden; Wayne State University, United States of America

## Abstract

Telomere length shortens with cellular division, and leukocyte telomere length is used as a marker for systemic telomere length. The hippocampus hosts adult neurogenesis and is an important structure for episodic memory, and carriers of the apolipoprotein E ε4 allele exhibit higher hippocampal atrophy rates and differing telomere dynamics compared with non-carriers. The authors investigated whether leukocyte telomere length was associated with hippocampal volume in 57 cognitively intact subjects (29 ε3/ε3 carriers; 28 ε4 carriers) aged 49–79 yr. Leukocyte telomere length correlated inversely with left (*r_s_* = −0.465; *p* = 0.011), right (*r_s_* = −0.414; *p* = 0.025), and total hippocampus volume (*r_s_* = −0.519; *p* = 0.004) among *APOE* ε3/ε3 carriers, but not among ε4 carriers. However, the ε4 carriers fit with the general correlation pattern exhibited by the ε3/ε3 carriers, as ε4 carriers on average had longer telomeres and smaller hippocampi compared with ε3/ε3 carriers. The relationship observed can be interpreted as long telomeres representing a history of relatively low cellular proliferation, reflected in smaller hippocampal volumes. The results support the potential of leukocyte telomere length being used as a biomarker for tapping functional and structural processes of the aging brain.

## Introduction

Telomeres are stretches of repetitive non-coding sequences, constituting the ends of linear chromosomes. As cells divide, the process of chromosomal replication inevitably leads to telomere shortening due to the end-replication problem. If a critical length is reached, the cell will either undergo apoptosis or enter a state of replicative senescence [1], [2]. This means that telomere length (TL) carries information concerning both replicative history and potential; properties which have generated a great deal of interest for telomere research. Leukocyte TL has generally been accepted as a measurement of systemic TL and has in the last decade been found to be associated with several age-related disorders and attributes; e.g., cardiovascular disease, cancer, and dementia [3], [4], [5].

Apolipoprotein E (APOE) is a polymorphic gene with three common alleles: ε2, ε3, and ε4, with typical population frequencies of approximately 5–10%, 70–85% and 10–25%, respectively. The *APOE* ε4 allele is a well-established genetic risk factor for Alzheimer's disease (AD) and influences episodic memory negatively in aging individuals, even in the absence of AD or mild cognitive impairment [6], [7]. We have previously shown that among middle-aged non-demented subjects, leukocyte TL was longer among *APOE* ε4 carriers compared with non-ε4 carriers, and further that worse episodic memory was associated with longer leukocyte TL among ε4 carriers [8]. Episodic memory depends to a large degree on the hippocampus, and several studies have found volumetric differences of the hippocampus between carriers and non-carriers of the *APOE* ε4 allele. Smaller hippocampi among ε4 carriers have been most consistently found among AD patients, but have also been observed among cognitively healthy individuals [9], [10]. Hippocampal memory functions are among adults typically found to be positively correlated to the volume of this brain region [11]. Cell turnover in the hippocampus is different with regards to most other regions in the brain as it is one of the few sites to host adult neurogenesis, believed to be important for learning and memory processes stemming from the hippocampus [12], [13]. This process of cellular proliferation allows for the possibility of telomere involvement, in its capacity as a determinant of replicative potential.

With the aforementioned as background, we set out to explore the potential relationship between hippocampal volume, as determined by magnetic resonance imaging (MRI), and leukocyte TL in both carriers and non-carriers of the *APOE* ε4 allele. The sample was drawn from the Betula study on memory and aging, located in northern Sweden, and consisted of 57 cognitively intact subjects, out of whom 29 carried the ε3/ε3 genotype, and 28 carried the ε4 allele.

## Results

General characteristics of the subjects participating in this study can be seen in Table 1. The mean age of the participants was 65.8 yr (SD = 8.2; ranging from 49 to 79), with 63% being females (age, males = 61.7 yr, SD = 8.9; females = 61.5 yr, SD = 8.0; *p* = 0.935). There were no significant differences between the 29 *APOE* ε3/ε3 and 28 *APOE* ε4 carriers regarding age, education, gender distribution, or any of the health parameters. Age-adjusted leukocyte TL was significantly longer among ε4 carriers (mean difference of 351 bp, *p* = 0.039), consistent with observations in the sample of which the current sample is part of [8]. TL decreased with increasing age (17 bp/year) in the full sample, but non-significantly (*p* = 0.118). Hippocampus volume (total) among ε3/ε3 carriers showed a negative correlation with age (*r_s_* = −0.404, *p* = 0.030), but there was no association between age and hippocampus volume among ε4 carriers (*r_s_* = −0.054 *p* = 0.783). Hippocampal volumes tended to be smaller among ε4 carriers, but the differences were not statistically significant (Table 1). (For more detailed analysis on hippocampal structural data in this sample, see Lind et al. 2006a.)

**Table 1:** General characteristics of study participants.

	Full Sample (*n* = 57)	*APOE* ε3/ε3 carriers (*n* = 29)	*APOE* ε4 carriers (*n* = 28)	*p*
Age, years (SD)	61.6 (8.2)	62.1 (8.5)	61.1 (8.1)	0.664
Gender, male/female (% male)	21/36 (37)	11/18 (38)	10/18 (36)	0.862
Education, years (SD)	10.4 (3.4)	10.2 (3.3)	10.6 (3.5)	0.631
BMI, kg/m^2^ (SD)	26.1 (3.2)	26.2 (3.2)	25.9 (3.3)	0.718
Smokers, *n* (%)	13 (23)	6 (21)	7 (25)	0.698
Systolic blood pressure, mmHg (SD)	143.2 (16.9)	143.6 (15.2)	142.7 (18.8)	0.835
Diastolic blood pressure, mmHg (SD)	82.4 (9.3)	82.9 (9.5)	81.9 (9.2)	0.667
Hypertension, *n* (%)^a^	34 (60)	18 (62)	16 (57)	0.705
Heart disease, *n* (%)	11 (19)	6 (21)	5 (18)	0.786
Diabetes (type 2), *n* (%)	2 (4)	1 (3)	1 (4)	0.980
Prescription drugs (SD)	1.2 (1.4)	1.1 (1.3)	1.3 (1.5)	0.702
MMSE score (SD)	28.1 (1.6)	27.9 (1.8)	28.2 (1.5)	0.571
Left hippocampus volume, mm^3^ (SD)^b^	2757 (284)	2750 (274)	2757 (288)	0.931
Right hippocampus volume, mm^3^ (SD)^b^	2895 (356)	2959 (371)	2827 (333)	0.162
Total hippocampus volume, mm^3^ (SD)^b^	5652 (565)	5709 (587)	5583 (537)	0.403
Leukocyte telomere length, bp (SD)^c^	5573 (644)	5401 (537)	5752 (705)	0.039

All values are means unless otherwise stated. MMSE, Mini-mental state examination; SD, standard deviation.

a Blood pressure ≥140/90 or taking antihypertensive medication.

b Adjusted for body (and head) size and age.

c Adjusted for age.

**Figure 1:**
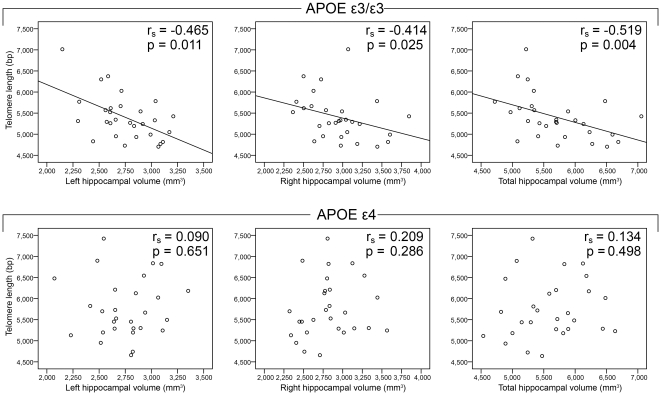
Correlations between leukocyte TL and hippocampal volume among *APOE* ε3/ε3 and *APOE* ε4 carriers. Leukocyte TL was adjusted for age. Hippocampal volume was adjusted for body (and head) size and age. Regression lines only shown for significant Spearman correlations.

Analyzing leukocyte TL (adjusted for age) and hippocampus volume (adjusted for age and body size) showed that among ε3/ε3 carriers, TL was significantly inversely correlated with hippocampus volume (Fig. 1); with left (*r_s_* = −0.465, *p* = 0.011), right (*r_s_* = −0.414, *p* = 0.025) as well as total volume (*r_s_* = −0.519, *p* = 0.004). No significant correlations between hippocampus volume and leukocyte TL were found among the ε4 carriers (left, *r_s_* = 0.090, *p* = 0.651; right, *r_s_* = 0.209, *p* = 0.286; total, *r_s_* = 0.134, *p* = 0.498; Fig. 1). Stratifying ε3/ε3 carriers by gender revealed similar, but non-significant, correlation coefficients among men (*n* = 11) and women (*n* = 18); indicating that both genders contributed to the observed correlation (males: left, *r_s_* = −0.391, *p* = 0.235; right, *r_s_* = −0.391, *p* = 0.235; total, *r_s_* = −0.427, *p* = 0.190; women: left, *r_s_* = −0.377, *p* = 0.123; right, *r_s_* = −0.340, *p* = 0.168; total, *r_s_* = −0.445, *p* = 0.064). Among ε4 carriers, no significant correlations were observed after stratifying for gender (males, *n* = 10: left, *r_s_* = 0.285, *p* = 0.425; right, *r_s_* = 0.261, *p* = 0.467; total, *r_s_* = 0.152, *p* = 0.676; women, *n* = 18: left, *r_s_* = 0.023, *p* = 0.929; right, *r_s_* = 0.326, *p* = 0.186; total, *r_s_* = 0.226, *p* = 0.367). Raising the MMSE inclusion criteria from ≥24 to ≥26, leading to exclusion of 5 ε3/ε3 and 5 ε4 carriers, did not alter the significances of the correlations (ε3/ε3: left, *r_s_* = −0.463, *p* = 0.023; right, *r_s_* = −0.601, *p* = 0.002; total, *r_s_* = −0.577, *p* = 0.003; ε4: left, *r_s_* = 0.102, *p* = 0.644; right, *r_s_* = 0.278, *p* = 0.199; total, *r_s_* = 0.194, *p* = 0.376). Considering the documented effect of age on both TL and hippocampal volume, age is a significant confounder in the analyses. Not taking the confounding effect of age and body/head size into account, i.e., performing the analyses completely unadjusted, weakened the correlations among ε3/ε3 carriers and made them non-significant (ε3/ε3: left, *r_s_* = −0.299, *p* = 0.108; right, *r_s_* = −0.247, *p* = 0.188; total, *r_s_* = −0.310, *p* = 0.096; ε4: left, *r_s_* = 0.215, *p* = 0.271; right, *r_s_* = 0.257, *p* = 0.186; total, *r_s_* = 0.226, *p* = 0.248); showing that for TL to be significantly associated with hippocampal volume among ε3 homozygotes, age has to be taken into account (correlations among ε3/ε3 carriers remained significant if age, but not body/head size, was adjusted for). Analysis for further confounding did not reveal any confounding of the correlation between TL and hippocampus volume by any of the health parameters listed in Table 1.

## Discussion

In the present study, we investigated whether leukocyte TL had any relationship with hippocampus volume among 57 non-demented individuals, aged 49 to 79 yr. In our sample of 29 ε3/ε3 carriers, TL was inversely correlated with hippocampus volume. Among the 28 ε4 carriers, no correlation was present; although, as a group the ε4 carriers fit with the correlation pattern found among ε3/ε3 carriers as they in general had longer TL and smaller hippocampi, compared with ε3/ε3 carriers.

Hippocampal volumes tended to be smaller among *APOE* ε4 carriers, but not significantly. However, as shown by Lind et al. [9], ε4 carriers in this sample aged 65 yr and below had significantly smaller right hippocampal volumes compared with ε3/ε3 carriers in the same age span. Other studies have reported similar observations, finding that the ε4 allele is associated with smaller hippocampi and increased hippocampal atrophy rates among ε4 carriers, compared with non-carriers [10], [14]. This is interesting in context of our findings, as the suppressive influence the ε4 allele appears to have on the hippocampus volume might explain the discrepancy in our findings: that of no association among ε4 carriers between TL and hippocampus volume while at the same time observing a moderate to strong corresponding association among ε3/ε3 carriers. Possibly related to this ε4 effect is the by us previously postulated hypothesis of an anti-proliferative, TL conserving, effect among middle-aged ε4 allele carriers [8]. The ε4 allele is associated with immunomodulatory effects, influencing both innate and adaptive immune responses, and it has been suggested that aberrant immunomodulatory activities may be associated with the role of *APOE* ε4 in AD [15]. Such effects can potentially influence both leukocyte TL and hippocampal volume, and thus alter the relationship between them. Also, it may be that a weaker association is present among ε4 carriers but that we lacked sufficient power to detect it.

In contrast to our findings, Grodstein et al. [16] reported a positive relationship between leukocyte TL and hippocampus volume. However, their sample was smaller, older (mean age = 79.2), only females, not taking *APOE* genotype into account, and included 7 (out of 26) participants with mild cognitive impairment (MCI). Considering that they also reported data showing that their participants with MCI had both smaller hippocampi and shorter TL (compared with the cognitively intact subjects), the inclusion of these subjects in their data analysis on the relationship between TL and hippocampal volume might to a large extent have explained their finding of a positive correlation. Our sample included no MCI cases, only cognitively healthy subjects.

Several studies that have compared TLs between different tissues in the same individuals have observed correlations between them; i.e., individuals with relatively long leukocyte TL have relatively long TLs in other tissues as well, although not necessarily of similar lengths in absolute terms [5], [17], [18]. Together with our findings, this suggests that non-demented ε3/ε3 carriers with relatively small hippocampi and long leukocyte TLs may also have relatively long TLs in hippocampal cells. To our knowledge, only one study so far has measured TL in hippocampal tissue. Thomas et al. [19] analyzed samples taken from AD patients (with unknown *APOE* status) and they found their hippocampal telomeres to be on average 49% longer compared with hippocampal tissue from age-matched non-demented subjects. Although their paper did not contain any volumetric information regarding the hippocampi which were sampled, a consistent finding among AD patients is the exhibition of smaller hippocampi compared with those of age-matched controls [20]. Under this assumption, their findings are in line with our observed inverse relationship between leukocyte TL and hippocampus volume. In addition to measuring hippocampal TL, Thomas et al. also measured leukocyte TL in Alzheimer's cases (and found them to be shorter compared with controls), but this was unfortunately not the same cases as they measured hippocampal TL in [19]. Therefore, the differences and correlations between leukocyte TL and hippocampal TL in AD patients in the study by Thomas et al is unknown.

Seeing as TL to some degree reflects historical proliferative activity, the finding by Thomas et al. [19] may be indicative of a relatively low level of cellular replication in the hippocampus of AD patients. A low activity of cellular division can in general be expected to coincide with a lower volume of the tissue in question. Further, cellular replication in the hippocampus includes adult neurogenesis, a process believed to be of importance for memory and learning and shown to be reduced among AD patients [12]. If the finding of Thomas et al. [19] indeed reflects a lower degree of hippocampal neurogenesis, and if hippocampal TL is correlated with leukocyte TL, then it can be hypothesized that leukocyte TL would be inversely associated with hippocampal functions influenced by adult neurogenesis. In line with this hypothesis is our recently published findings, where we presented results showing an inverse relationship between leukocyte TL and episodic memory tasks, a memory system which depends on the hippocampus for proper functioning. This inverse relationship was significant among ε4 carriers, and borderline significant among ε3/ε3 carriers [8].

TL was significantly longer among carriers of the APOE ε4 allele, as reported in our previous study [8] (which also included the individuals taking part in this study). As mentioned above, we speculated that this may be due to an anti-proliferative, TL conserving, effect exerted by the ε4 allele. It is also possible, as the ε4 allele has shown immunomodulatory properties and that different subsets of leukocytes to some degree have different TL [21], [22], [23], that the ε4 allele influences leukocyte subset ratios and subsequently leukocyte TL. However, there are, to our knowledge, no studies indicating that the APOE genotype influences leukocyte subset ratios. The lack of information regarding leukocyte subsets ratios is a potential limitation of the study.

In summary, we hypothesize that proliferative activity is the key to our observed inverse relationship between leukocyte TL and hippocampus volume among *APOE* ε3/ε3 carriers, as well as part of the explanation as to why ε4 carriers as a group fit with the same inverse relationship. Low activity of proliferation can explain long telomeres coinciding with relatively small hippocampal volumes and fits with our previous findings linking long leukocyte TL with poor performance of episodic memory, a memory system believed to be dependent on adult neurogenesis taking place in the hippocampus. This hypothesis is also compatible with what was found by Thomas et al. [19]. Our findings warrant further studies to replicate our observations and further expand upon them, as they show the potential of TL acting as a biomarker for processes within the brain related to both functional and structural changes. Extended knowledge about details of TL and TL attrition rates in both the brain and leukocytes, among both healthy and diseased, will potentially provide valuable insight into the processes governing the aging of the brain, and how these possibly can be tapped by measurement of TL.

## Materials and Methods

### Ethics Statement

All subjects gave written informed consent, all clinical investigations were conducted according to the principles expressed in the declaration of Helsinki, and the study was approved by The Regional Ethical Review Board in Umeå, Sweden.

### Study participants

This study included a sample of 57 individuals (21 males, 36 females), aged 49 to 79; all recruited from the Betula study. The Betula study is an ongoing prospective cohort study initiated in 1988 aimed at exploring various aspects of memory, health and aging. To date, approximately 4500 participants have been enrolled in the Betula study, all randomly selected from the population registry of Umeå municipality, Sweden. The study population has been validated to conform well with the general population of northern Sweden. Exclusion criteria for enrollment into the Betula project were dementia, mental retardation, serious visual or auditory handicaps, not having Swedish as a mother tongue, or any other feature that would compromise the ability to comply with the study protocol (for full details regarding the Betula study, see [24], [25]). Sixty subjects were recruited from the Betula pool of subjects for participation in a MRI-based multiple outcome study. Thirty subjects were *APOE* ε3/ε3 carriers, 20 ε3/ε4 carriers and 10 were ε4/ε4 carriers. *APOE* ε4 homozygotes were recruited initially. Following this, age-, gender-, and education-matched ε3/ε3 and ε3/ε4 carriers were recruited. All subjects were non-demented (Mini-Mental State Examination ≥24; [26]) and cognitively intact [25], [27]. 57 subjects were eligible for participation within the present study. This study sample of 57 individuals was also part of the larger sample studied in Wikgren et al. [8] and Lind et al. [9], [27]. For more details concerning this sample, see Lind et al. [27].

### Laboratory procedures

Leukocyte DNA was extracted from whole blood using standard procedures. All blood samples were drawn within a 5 year time-frame of the MRI examination. *APOE* genotyping was carried out using a one-stage PCR method, as previously described [7]. Leukocyte TL was measured using a quantitative real-time PCR as described earlier [28], [29]. In short, each DNA sample was amplified on two parallel 96-well PCR plates; one amplifying telomere repeats and the other amplifying a single copy gene (*β2-globin*). The ratio of the mean telomere repeat copy number to the mean single copy gene copy number (T/S ratio) as related to that of a reference sample (DNA from the cell line CCRF-CEM) reflects relative TLs. Relative TLs (measured in relative T/S ratios) were converted to telomere restriction fragment lengths (measured in base pairs) based on correlation data derived from samples measured using both quantitative real-time PCR and the conventional southern blot method. Plasma cotinine was measured on the IMMULITE® 2000 Immunoassay System (Siemens Medical Solutions Diagnostics, Los Angeles, CA). Cotinine is the major metabolite of nicotine, and measurement of it is the favored method of detecting use of tobacco products [30]. Measured plasma cotinine values (25 ng/mL was used as cutoff) together with self-reported tobacco products usage were used for categorizing individuals as smoker or non-smoker.

### Imaging methods

Magnetic resonance imaging was performed using a Philips Intera 1.5 T scanner (Philips Medical Systems, Netherlands). Results from structural scans carried out on this sample have been reported previously [9]. A total of 124 coronal slices (1.8 mm thickness) were acquired using a T1-weighted 3D gradient echo (TR = 24 ms, TE = 6 ms, flip angle = 35°, FOV = 180 mm×180 mm). Images were corrected for undesirable effects of head tilt, rotation, and pitch using Brain Image 5.2.5. To achieve isotropic voxel size, bicubic interpolation between the slices was performed using Matlab 6.1 (Mathworks Inc., MA, USA). The hippocampus was traced manually on the coronal slices by J.L. using NIH Image 1.20. All measurements were performed blinded to the characteristics of the study subjects. Further details concerning the imaging method and hippocampus volume determination are available in Lind et al. [9].

### Statistical analysis

Means were compared using the independent-samples *t*-test. Distributions of categorical variables were tested using the Pearson chi-square test. The rate of TL shortening with age was determined using linear regression. To avoid undue influence of outliers, and due to low number of participants in each *APOE* subgroup, Spearman's rho (non-parametric) was used to evaluate correlations between TL and hippocampus volume, and between age and hippocampus volume. TL was age adjusted, and body height was used to adjust hippocampal volumes for differences in body (and head) size via analysis of covariance [31]. Hippocampal volumes were also age-adjusted. P-values below 0.05 were considered significant. All statistical analyses were performed using PASW Statistics 18.0 (SPSS Inc., Chicago, IL).
